# Free-fatty acid receptor-1 (FFA1/GPR40) promotes papillary RCC proliferation and tumor growth via Src/PI3K/AKT/NF-κB but suppresses migration by inhibition of EGFR, ERK1/2, STAT3 and EMT

**DOI:** 10.1186/s12935-023-02967-x

**Published:** 2023-06-24

**Authors:** Priyanka F. Karmokar, Nader H. Moniri

**Affiliations:** 1 Department of Pharmaceutical Sciences, College of Pharmacy, Mercer University Health Sciences Center, Mercer University, 3001, Mercer University Drive, Atlanta, GA 30341 USA; 2 Department of Biomedical Sciences, School of Medicine, Mercer University Health Sciences Center, Mercer University, Macon, GA 31207 USA

**Keywords:** Free-fatty acids, Free-fatty acid receptors, FFA1, GPR40, Renal cell carcinoma, Papillary RCC

## Abstract

**Background:**

Papillary renal cell carcinoma (pRCC) is a highly metastatic genitourinary cancer and is generally irresponsive to common treatments used for the more prevalent clear-cell (ccRCC) subtype. The goal of this study was to examine the novel role of the free fatty-acid receptor-1 (FFA1/GPR40), a cell-surface expressed G protein-coupled receptor that is activated by medium-to-long chained dietary fats, in modulation of pRCC cell migration invasion, proliferation and tumor growth.

**Methods:**

We assessed the expression of FFA1 in human pRCC and ccRCC tumor tissues compared to patient-matched non-cancerous controls, as well as in RCC cell lines. Using the selective FFA1 agonist AS2034178 and the selective FFA1 antagonist GW1100, we examined the role of FFA1 in modulating cell migration, invasion, proliferation and tumor growth and assessed the FFA1-associated intracellular signaling mechanisms via immunoblotting.

**Results:**

We reveal for the first time that FFA1 is upregulated in pRCC tissue compared to patient-matched non-cancerous adjacent tissue and that its expression increases with pRCC cancer pathology, while the inverse is seen in ccRCC tissue. We also show that FFA1 is expressed in the pRCC cell line ACHN, but not in ccRCC cell lines, suggesting a unique role in pRCC pathology. Our results demonstrate that FFA1 agonism promotes tumor growth and cell proliferation via c-Src/PI3K/AKT/NF-κB and COX-2 signaling. At the same time, agonism of FFA1 strongly inhibits migration and invasion, which are mechanistically mediated via inhibition of EGFR, ERK1/2 and regulators of epithelial–mesenchymal transition.

**Conclusions:**

Our data suggest that FFA1 plays oppositional growth and migratory roles in pRCC and identifies this receptor as a potential target for modulation of pathogenesis of this aggressive cancer.

**Supplementary Information:**

The online version contains supplementary material available at 10.1186/s12935-023-02967-x.

## Background

Renal cell carcinoma (RCC) is one of the most common genitourinary cancers, accounting for 90% of all kidney cancers and having a high degree of mortality amongst the urogenital cancers [1]. Several unique subtypes of RCC are recognized, and exhibit distinct histological, molecular, and pathological characteristics, with clear cell RCC (ccRCC) being the most common, followed by papillary RCC (pRCC), which accounts for 15–25% of all cases and is characterized by aggressive metastasis and resistance to standard treatments that are otherwise generally effective towards ccRCC [2, 3].

A wealth of epidemiological and experimental evidence has shown that dietary fats, particularly free-fatty acids (FFA), can influence a variety of cancers through mechanisms that include regulation of cell structure, gene expression, energy utilization, and intracellular signaling [4–8]. Nearly 20 years ago, a family of cell-surface G protein-coupled receptors that are activated by FFA was discovered and shown to mediate many of the effects of dietary fats. The FFA receptor (FFAR) family includes FFA2 (GPR43) and FFA3 (GPR41), which are agonized by short-chain fatty acids such as acetate and butyrate, and FFA1 (GPR40) and FFA4 (GPR120), which are agonized by omega-3, -6 and -9 and other medium-to-long chained fatty acids [9, 10]. Emerging evidence shows that FFA receptors also are involved in positive or negative regulation of a myriad of oncogenic processes including cancer cell proliferation, metastasis, angiogenesis, and chemoresistance, as we and others have previously reviewed [11–14].

Recently, we reported the first evidence that FFAR may play functionally significant roles in RCC, as agonism of FFA4 was shown to stimulate pRCC migration and invasion, while at the same time inhibiting cell proliferation and tumor growth [15]. Here, we reveal for the first time that expression of FFA1 is increased in pRCC tissues from human tissue compared to patient-matched noncancerous adjacent kidney tissue, and that FFA1 expression is also associated with pathological progression of pRCC. Meanwhile, FFA1 expression seems to be lost as pathology of ccRCC increases and transcript and protein for the FFAR was found in pRCC cell line ACHN but not ccRCC cell lines. Using selective FFA1 agonists and antagonists, we assessed the mechanistic roles of FFA1 in pRCC cell proliferation, wound healing, migration, invasion, and tumor growth, which together characterize various cancerous outcomes. Our results demonstrate that FFA1 exhibits opposing effects on cell growth and motile activities. Moreover, the agonism of FFA1 regulates pRCC cell migration and invasion by affecting the elements of EGFR, ERK, STAT3, and EMT pathways. In addition, we reveal that FFA1-mediated serum-induced cell proliferation is regulated by c-Src/PI3K/AKT/NF-κB/COX-2 signaling. Taken together, our results indicate that FFA1 could be a unique potential target for the treatment of pRCC and our findings help to understand the molecular aspects of pRCC development and progression.

## Methods

### Chemicals and antibodies

GW9508, GW1100, AG1478, and celecoxib were purchased from Sigma-Aldrich (St. Louis, MO). AS2034178, GM6001, and Phorbol 12-myristate 13-acetate (PMA) were purchased from Tocris Bioscience (Minneapolis, MN). Human EGF protein was obtained from PeproTech US (Cranbury, NJ). MK-2206 dihydrochloride was purchased from Advanced Chem Blocks Inc (Hayward, CA). BAY 11-7082 was obtained from TCI America (Portland, OR). Mitomycin C was purchased from MP Biomedicals (Irvine, CA). Monoclonal antibodies recognizing phospho-p44/42 MAPK (pERK1/2) (Thr202/Tyr204) (9101), p44/42 MAPK (ERK-1/2) (9102), phospho-AKT (Thr308) (9275), phospho-AKT (Ser473) (4060), AKT (9272), phospho-EGFR (Tyr1068) (2234), EGFR (4267), phospho-STAT3 (Tyr705) (9145), STAT3 (9139), phospho-Src (Tyr416) (2101), Src (2109), NF-κB p65 (8242), PI3 Kinase p85α (13,666), MMP-9 (3852), COX-2 (4842), E-Cadherin (3195), N-Cadherin (13116), Vimentin (5741), and Fibronectin/FN1 (26,836) were purchased from Cell Signaling Technology (Danvers, MA). Anti-FFA1 antibody (ab236285) for IHC was purchased from Abcam (Waltham, MA) while polyclonal anti-FFA1 (NB100-1537) for immunoblotting was obtained from Novus Biologicals (Centennial, CO). Anti-β-actin antibody (sc-47778) was purchased from Santa Cruz Biotechnology (Dallas, TX). Goat anti-mouse and goat anti-rabbit HRP secondary antibodies were purchased from Thermo Fisher Scientific (Waltham, MA).

### Cell culture and maintenance

Human RCC cell lines ACHN, 786-O, and Caki-1 were initially a generous gift from Dr. Yehia Daaka (University of Florida College of Medicine, Gainesville, FL) and subsequently purchased from ATCC and cultured in RPMI 1640 supplemented with 10% fetal bovine serum (FBS) and 1% penicillin-streptomycin (PS). HEK-293 cell line (CRL-1573) was purchased from ATCC and maintained in DMEM containing 10% FBS and 1% PS. Cells are authenticated via STR profiling and mycoplasma tested prior to use. Cells were maintained in a humidified incubator containing 5% CO_2_ at 37 °C and all experiments were performed between passage number 3 and 10. To assess the role of β-arrestin, cells in 100 mm dishes were transfected with pcDNA3-β-arrestin-(319–418) (kind gift from Dr. Jeffrey Benovic, Thomas Jefferson University) or pcDNA3-β-arrestin-2-YFP (kind gift from Dr. Michel Bouvier, University of Montreal) using 2 µg of the respective plasmid and LipoD293 DNA transfection reagent (Signagen Laboratories, Fredrick, MD), according to the manufacturer’s instructions.

### Reverse transcription-polymerase chain reaction (RT-PCR)

Total RNA from RCC cells was extracted using Trizol reagent (Invitrogen, Carlsbad, CA) with deoxyribonuclease I (Invitrogen) and first-strand cDNA was synthesized using Omniscript (Qiagen, Germantown, MD) according to the manufacturer’s instructions. 300 ng of cDNA was utilized in PCR at 35 cycles, 10 s at 98 °C, 30 s at 55 °C, 60 s at 72 °C, and a final extension of 10 min at 72 °C. The primer sequences used to amplify targeted transcripts were: [FFA1], 5′-ACCTGCCCCCGCAGCTCTCCTTCG-3′ (forward), 5′-AGGACCCCTTCCCAAGTA-3′ (reverse); [GAPDH], 5′–ACCCCTTCATTGACCTCAACTAC-3′ (forward), 5′-ATGAGGTCCACCACCCTGTTGC-3′ (reverse).

### Cell proliferation assay

Subconfluent cells were starved in serum-free media for 24 h and subsequently seeded in 6-well plates at 5 × 10^4^ cells/well in RPMI-1640 medium containing 10% FBS. Cells were treated as indicated with either the FFA1/4 dual agonist GW9508 or the selective FFA1 agonist AS2034178, in the presence and absence of FFA1 antagonist GW1100 every 24 h for 6 days. In separate assays, cells were incubated with MK2206 (1 µM), celecoxib (0.5 µM), and BAY 11-7082 (1 µM), which selectively inhibit activation of AKT, COX-2, and NF-κB, respectively, 30 min prior to the addition of AS2034178. Cell counting was assessed every 24 h for 6 days using an automated TC20 cell counter (Bio-Rad, Hercules, CA) and trypan blue exclusion.

### Wound-healing assay

Subconfluent ACHN cells were seeded at 1.5 × 10^5^ cells/well and allowed to adhere for 24 h. Cells were starved for 24 h in serum-free media and treated with MMC (2.5 µg/mL) for 2 h to inhibit cell proliferation. A sterile pipette tip was used to make a scratch wound and media with appropriate agent(s) was added. Wound recovery, a measure of cell migration, was assessed 24 h later using an Echo Revolve R4 microscope (Discover Echo, San Diego, CA).

### Transwell assays

Migration and invasion was evaluated using 6.5 mm 24 well-transwell plates containing 8.0 μm pore polycarbonate membrane inserts and BioCoat Matrigel chambers, respectively (Corning Life Sciences, Corning, NY). Following 24 h of starvation, 2.5 × 10^4^ cells were added to upper chambers with appropriate agent(s), and 700 µL of RPMI 1640 media containing 10% FBS was added to the lower chambers. After 16 h at 37 °C, a cotton-tipped swab was used to remove cells from the top of the membrane and migrating cells attached to the bottom of the membrane were fixed using methanol, stained using 0.2% crystal violet solution, allowed to air dry and imaged and counted using an Echo Revolve microscope.

### Immunoblotting

Immunoblotting was performed as we have previously described [15–17]. Representative blots are shown for experiments performed 3–5 times as described in the figure legends. The respective graphs quantify the relative expression as a percentage of the vehicle-treated control and are expressed as mean ± SD of all *n* performed.

### Immunohistochemistry

Formalin fixed paraffin embedded tissue sections of human ccRCC and pRCC samples and patient-matched normal controls were provided by Dr. Viraj Master (Department of Urology and Winship Cancer Institute, Emory University School of Medicine, Atlanta, GA). Sections were deparaffinized and rehydrated through descending series of graded ethanol, and boiled at 100 °C for 20 min in sodium citrate buffer (10 mM Sodium Citrate, 0.05% Tween 20, pH 6.0), followed by two washes and blocking in 10% horse serum with 1% BSA. After blocking, sections were incubated overnight with anti-FFA1 antibody (1:400) at 4 °C, washed three times with gentle agitation, and further blocked with 0.3% H_2_O_2_ to block endogenous peroxidase activity, followed by incubation with goat anti-rabbit IgG H&L (HRP) secondary antibody (1:20,000) at room temperature for 1 h. For visualization, sections were incubated with DAB chromogen for 10 min and counterstained using Mayer’s hematoxylin. Images were captured using Echo Revolve brightfield microscope.

### In vivo tumorgenicity assays

Three to four-week-old male, homozygous athymic nude mice were purchased from Charles River Laboratories (Wilmington, MA) (strain code 490), randomized, and acclimated to the environment for 1 week. All mice were housed under pathogen-free conditions with a controlled temperature (25 °C) and humidity (60%), in a 12 h light/dark cycle and were fed sterilized chow and autoclaved water. All animal procedures complied with the NIH Guidelines for the Care and Use of Laboratory Animals and were approved by the Mercer University Institutional Animal Care and Use Committee. Tumors were implanted into the right flanks using 2 × 10^6^ ACHN cells in 100 µL sterile PBS [18–20]. One week after the implantation, mice were randomly divided into four treatment groups (*n* = 6 each): vehicle-control, AS2034178 (10 mg/kg), GW1100 (10 mg/kg), and AS2034178 + GW1100 [20], each dissolved in sterile PBS containing PEG 400 (8%), DMSO (4%), and Tween 80 (8%) and injected intraperitoneally daily for 28 days. Body weight and tumor diameter were recorded every 3 days and tumor volume was calculated using the following formula (tumor volume = length × width^2^ × 0.52). Mice were euthanized 24 h following the final treatment and tumors were surgically excised, measured, weighed, imaged, and stored at – 80 °C for future experiments.

### Data analysis

Data were expressed as the mean ± SD of at least three independent experiments. Statistical significance was determined by paired *t*-test, except for the proliferation experiments where two-way analysis of variance (ANOVA) followed by Tukey’s multiple comparisons post hoc test was used due to multiple variables. Values of *p* < 0.05 were defined a priori as statistically significant and reported *p* values are accompanied by Cohen’s d value as a measure of the effect size to convey practical significance. For immunoblots, representative blots are shown while graphed representation contains statistical analysis for all *n* performed, as indicated in the text and figure legends. All analyses were performed using Prism software (GraphPad Software, San Diego, CA).

## Results

### Increased expression of FFA1 in pRCC and ccRCC tissues

**Fig. 1:**
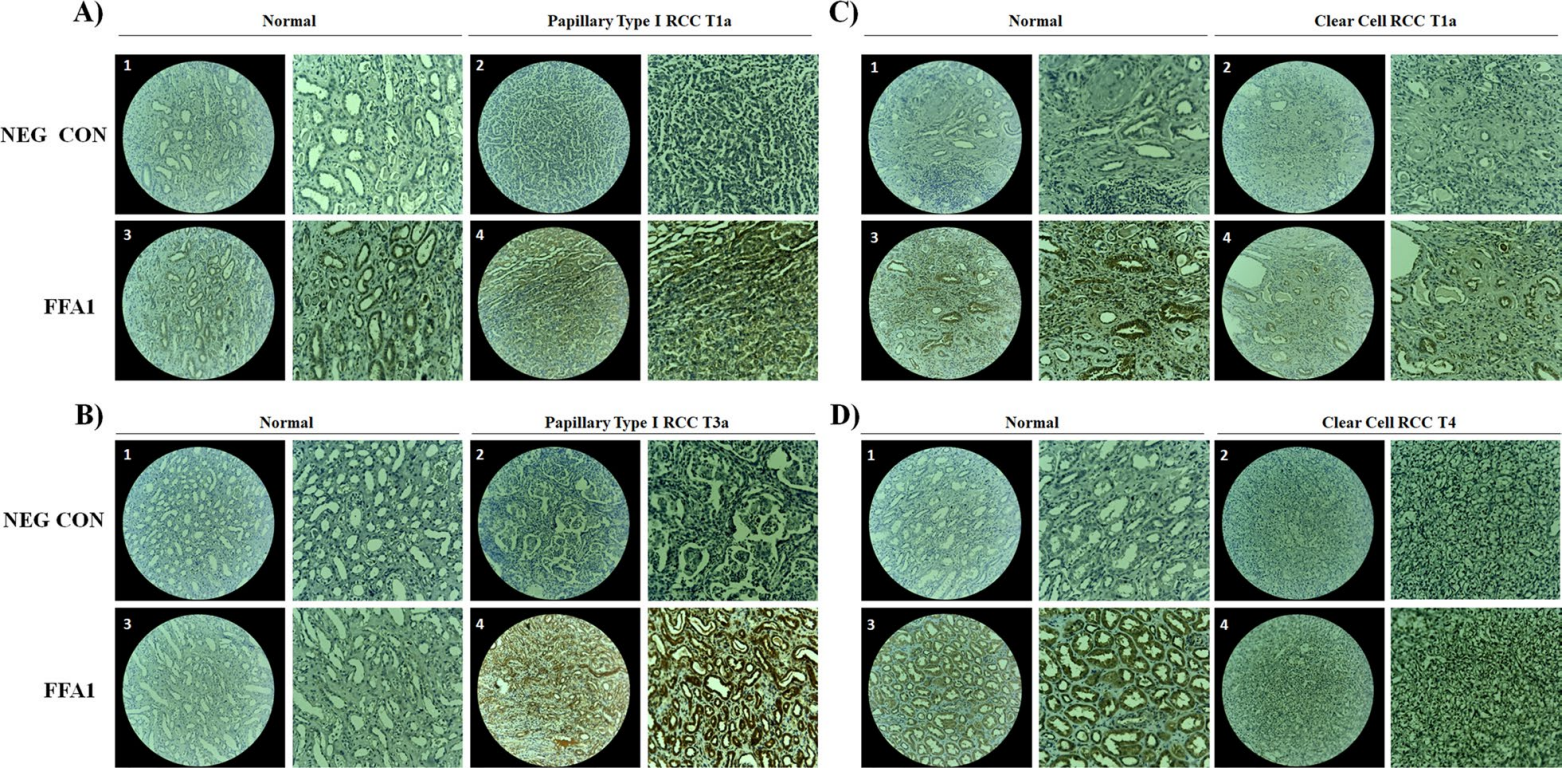
FFA1 expression is increased in pRCC and visibly lost in ccRCC. Expression of FFA1 was determined in normal (noncancerous) tissue compared with adjacent patient-matched type 1 pRCC in stage T1a (**A**), type 1 pRCC in stage T3a (**B**), ccRCC in stage T1a (**C**), and ccRCC in stage T4 (**D**). For each, representative images were captured using an Echo Revolve microscope with the left image (numbered) representing the 20× magnification field and the right image representing 170% zoom to the center of the field. Panels 1–2 correspond to the negative control condition, which lacks the FFA1 antibody. Panels 3–4 represent specific staining in the presence of the FFA1 antibody. **A** Expression of FFA1 is visibly higher in stage T1a pRCC (**A4**) compared to patient-matched adjacent normal tissue (**A3**). **B** Expression of FFA1 is visibly higher in stage T3a pRCC (**B4**) compared to patient-matched normal tissue (**B3**). Importantly, the expression of FFA1 is increased in stage T3a (**B4**) compared to stage T1a (**A4**). **C** Expression of FFA1 is visibly lower in stage T1a ccRCC (**C4**) compared to patient-matched adjacent normal tissue (**C3**). **D** Expression of FFA1 is visibly absent in stage T4 ccRCC (**D4**) compared to patient-matched normal tissue (**D3**)

To investigate the potential importance of FFA1 in pRCC, we examined and qualitatively compared the level of expression of FFA1 at different pathological stages between paraffin-embedded pRCC and ccRCC tissues and their respective patient-matched normal kidney tissues. Here, we show for the first time that FFA1 expression is visibly elevated in stage T1 and T3 pRCC compared to matched non-cancerous tissue (Fig. 1A, B, 3–4). Importantly, FFA1 staining was markedly increased in stage T3a pRCC (Fig. 1B4) compared to stage T1a (Fig. 1A4), suggesting that FFA1 is positively correlated with pRCC progression. On the other hand, FFA1 immunostaining was comparatively lower in ccRCC tissue samples than that in patient-matched adjacent noncancerous tissues, with the normal luminal expression visibly decreased in ccRCC tissue (Fig. 1C3-4). Moreover, FFA1 immunostaining was nearly undetectable in advanced T4 stage ccRCC (Fig. 1D4) compared to the relatively stronger luminal immunostaining in matched normal tissue sections (Fig. 1D3), suggesting that FFA1 expression is negatively associated with pathologic progression in ccRCC.

### FFA1 mRNA and protein are detected in the ACHN papillary RCC cell line

To begin to study the role of FFA1 in RCC cells, we first examined the expression of FFA1 transcripts by RT-PCR analysis in RCC cell lines, including the ccRCC cell lines 786-O and Caki-1 and the pRCC cell line ACHN. As shown in Additional file 1: Fig. S1, FFA1 transcript (Additional file 1: Fig. S1A) and protein (Additional file 1: Fig. S1B) were expressed in ACHN cells, while consistent with IHC expression data from human subjects in Fig. 1, we were unable to detect FFA1 in either of the primary and metastatic clear cell RCC cell lines 786-O and Caki-1, respectively (Additional file 1: Fig. S1A). These data suggest that expression of FFA1 may be limited to the papillary but not the clear cell subtype of RCC.

### FFA1 agonism promotes pRCC cell proliferation

**Fig. 2:**
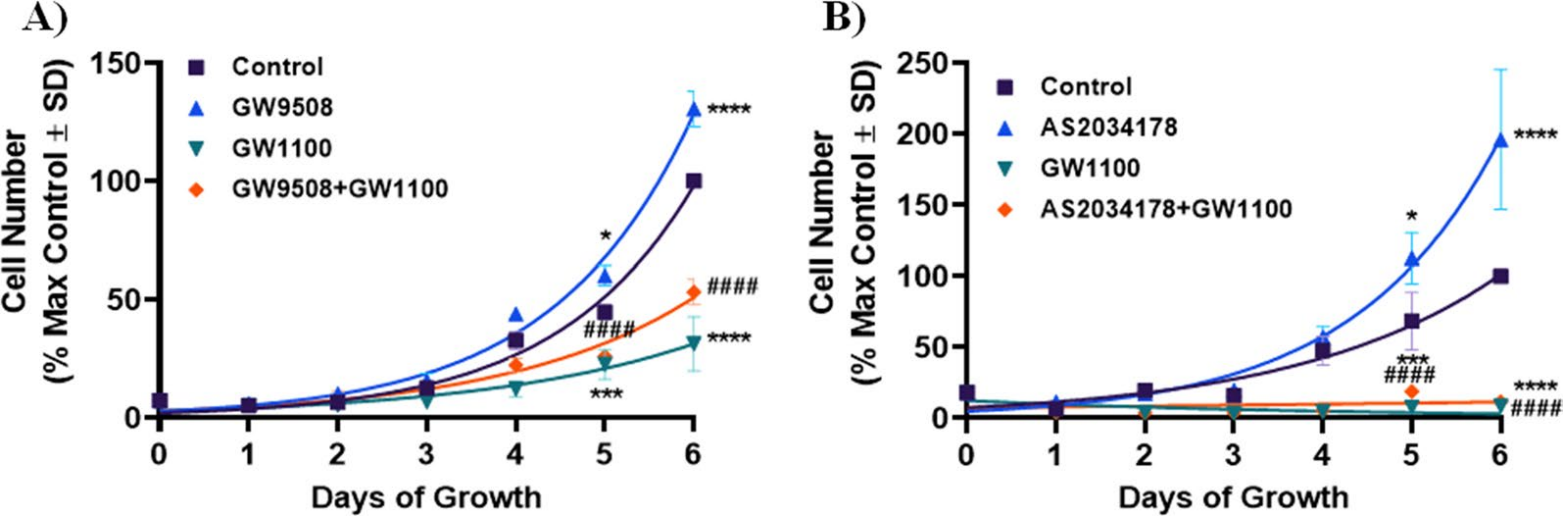
FFA1 regulates ACHN cell proliferation. **A** The FFA1/4 agonist GW9508 (10 µM) induced significant increases in cell proliferation on days 5 (*p* < 0.05; d = 2.4, versus control) and 6 (*p* < 0.0001; d = 3.3, versus control). This effect of GW9508 was significantly inhibited by the selective FFA1 antagonist GW1100 (10 µM) on days 5 (*p* < 0.0001; d = 6.3, versus GW9508) and 6 (*p* < 0.0001; d = 6.38, versus GW9508). Compared to the vehicle-treated control condition, GW1100 alone significantly inhibited cell proliferation at days 5 (*p* < 0.001; d = 9.9, versus control) and 6 (*p* < 0.0001; d = 4.9, versus control). **B** The selective FFA1 agonist AS2034178 significantly increased cell proliferation on days 5 (*p* < 0.05; d = 1.4, versus control) and 6 (*p* < 0.0001; d = 1.6, versus control) compared to vehicle-treated cells, and the overall effect of AS2034178 was noticeably greater than that of the dual FFA1/4 agonist GW9508. GW1100 completely inhibited the effects of AS2034178 at day 5 (*p* < 0.0001; d = 4.1, versus AS2034178) and 6 (*p* < 0.0001; d = 3.1, versus AS2034178) of growth, while again, when used alone, GW1100 significantly inhibited cell proliferation at days 5 (*p* < 0.001; d = 38.9, versus control) and 6 (*p* < 0.0001; d = 16.0, versus control). Differences between groups were evaluated by two-way analysis of variance (ANOVA) followed by Tukey’s multiple comparisons post-hoc test. Graphs depict combined replicate data from three independent experiments each performed in triplicate. * denotes *p* < 0.05, *** denotes *p* < 0.001, and **** denotes *p* < 0.0001 versus the control condition. #### denotes *p* < 0.0001 versus the AS2034178-treated condition

Next, we wished to determine if FFA1 could modulate in vitro proliferation of pRCC cells and our initial experiments utilized the dual FFA1/FFA4 agonist GW9508, which exhibits 75-fold selectivity for FFA1 [21]. Treatment of ACHN cells with GW9508 (10 µM) led to a significant increase in serum-induced cell proliferation on days 5 and 6 (*p <* 0.05; d = 2.4 and *p <* 0.0001; d = 3.3 versus control, respectively) (Fig. 2A). The increase in cell growth in response to GW9508 at days 5 and 6 was markedly blocked by the selective FFA1 antagonist GW1100 (10 µM) [21] (*p <* 0.0001; d = 6.3 and *p <* 0.0001; d = 6.38 versus GW9508, respectively) (Fig. 2A). When used alone, GW1100 significantly inhibited cell proliferation at days 5 and 6 compared to the vehicle-treated control condition (*p <* 0.001; d = 9.9 and *p <* 0.0001; d = 4.9 versus control, respectively) (Fig. 2A). To confirm these effects were indeed mediated by FFA1 agonism, we utilized the selective FFA1 agonist AS2034178, which lacks functional activity at other FFA receptors, including FFA4 [22]. Treatment of ACHN cells with AS2034178 (10 µM used throughout) significantly increased cell proliferation on days 5 and 6 compared to vehicle-treated cells (*p <* 0.05; d = 1.4 and *p <* 0.0001; d = 1.6 versus control, respectively) (Fig. 2B). Interestingly, the effects seen with this agonist were higher than that seen with GW9508, suggesting that removal of the FFA4-acting component facilitates greater proliferation, consistent with our recent report that FFA4 agonism inhibits cell proliferation [15]. As seen with GW9508, GW1100 completely inhibited the effects of AS2034178 at days 5 and 6 of growth (*p <* 0.0001; d = 4.1 and *p <* 0.0001; d = 3.1 versus AS2034178, respectively), and GW1100 alone also significantly inhibited cell proliferation at days 5 and 6 (*p <* 0.001; d = 38.9 and *p <* 0.0001; d = 16.0 versus control, respectively) (Fig. 2B). Taken together, these results demonstrate that agonism of FFA1 increases ACHN cell proliferation.

### FFA1 regulates cell migration and wound healing

**Fig. 3:**
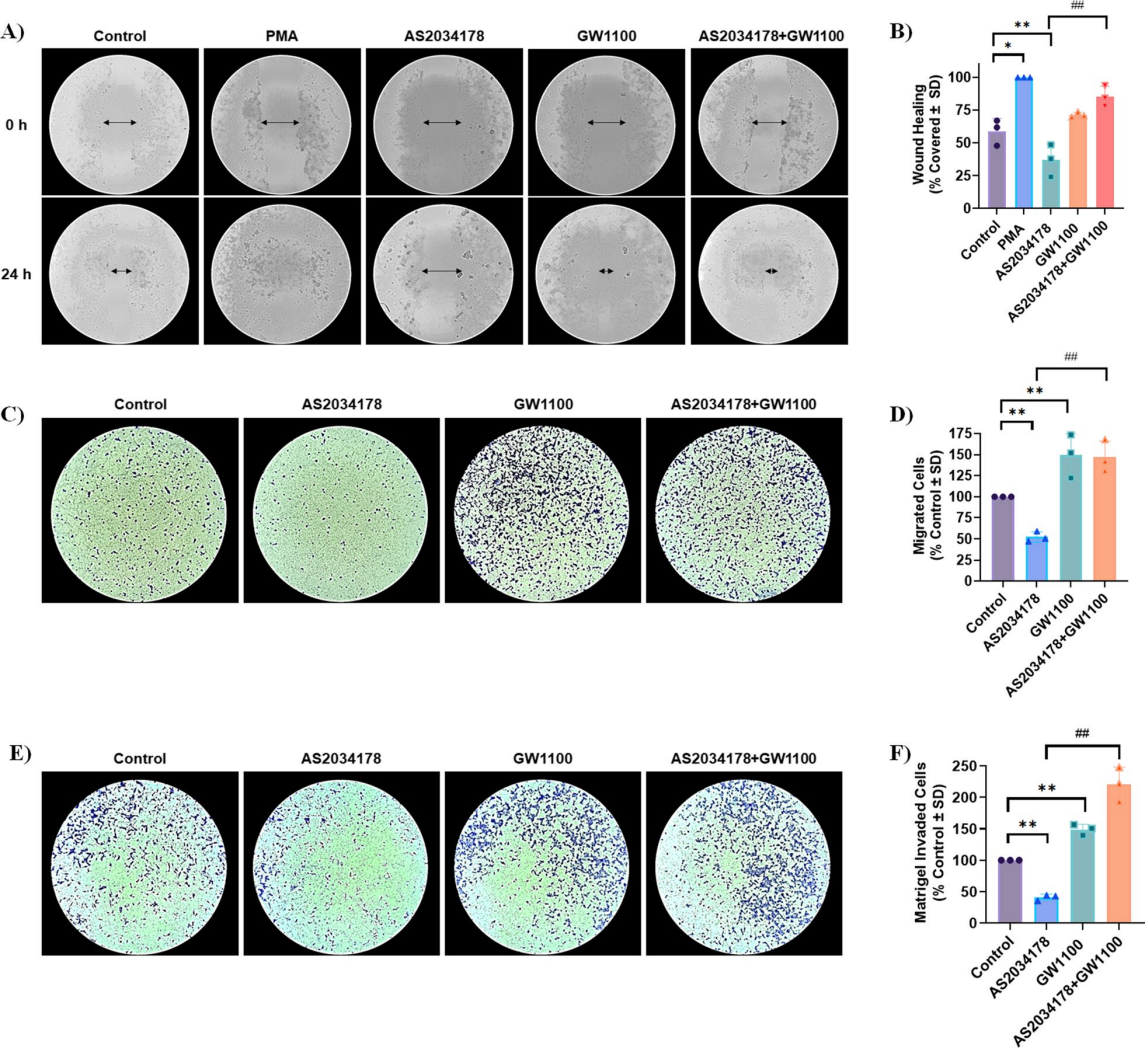
FFA1 regulates the migratory and invasive capacity of ACHN pRCC cells. **A**, **B** The effects of FFA1 on the migration of ACHN pRCC cells were evaluated by wound scratch assay. All conditions are in the presence of MMC to inhibit proliferation and MMC had no effect alone (not shown). PMA (1 µM) was used as positive control while media without serum was used as a negative control (not shown). PMA treated wounds closed fully after 24 h (*p *< 0.05; d = 5.9, versus control), while AS2034178 (10 µM) treated cells had significant wider wounds than control cells (*p* < 0.01; d = 5.9, versus control). The effects of AS2034178 were fully inhibited by GW1100 (*p* < 0.01; d = 4.7, versus AS2034178 alone), while GW1100 alone also caused near-full closure of the wound. Statistical significance was determined by paired t-test. * denotes *p* < 0.05 and ** denotes *p* < 0.01 compared to the vehicle-treated control condition, while ## denotes *p* < 0.01 compared to the AS2034178-treated condition. **C**, **D** The role of FFA1 in migration was also confirmed using in vitro transwell cell migration assays. AS2034178 (10 µM) facilitated significantly lower migration compared to vehicle-treated controls (*p* < 0.01; d = 11.1, versus control), while GW1100 significantly inhibited this effect (*p* < 0.01; d = 6.7, versus AS2034178). GW1100 alone demonstrated significantly higher migration compared to control (*p* < 0.01; d = 2.7, versus control). Graphs depict combined replicate data from three independent experiments. Statistical significance was determined by paired t-test. ** denotes *p* < 0.01 versus the control condition, while ## denotes *p* < 0.01 versus the AS2034178-treated condition. **E**, **F** The invasive capacity of pRCC cells was evaluated by a Matrigel-coated transwell assay. AS2034178 (10 µM) facilitated significantly lower invasion through the matrix compared to vehicle-treated controls (*p* < 0.01; d = 17.3, versus control), and GW1100 significantly inhibited this effect (*p* < 0.01; d = 15.6, versus AS2034178). GW1100 alone demonstrated significantly higher migration compared to control (*p* < 0.01; d = 8.0, versus control). The graph depicts combined replicate data from three independent experiments. Statistical significance was determined by paired t-test. ** denotes *p* < 0.01 versus the control condition and ## denotes p < 0.01 versus the AS2034178-treated condition

Since ACHN cells are the only well-characterized metastatic papillary RCC cell line, we wished to determine if FFA1 modulates the migratory capacity of ACHN pRCC cells as a measure of metastatic potential. First, we assessed the effects of FFA1 agonism and antagonism on directional cell migration using wound-healing assays, and in order to discount the contribution of cell proliferation in these results, all assays were performed in the presence of mitomycin C (MMC; 2.5 µg/mL for two h prior to assay) to inhibit cell proliferation, and for 24 h, a period at which FFA1 agonism had no influence on proliferation (Fig. 2A, B). There were no visible differences seen after 24 h between untreated cells and those treated with MMC (data not shown). The phorbol ester phorbol 12-myristate 13-acetate (PMA) was used as a positive control, and PMA-treated wounds closed fully after 24 h (Fig. 3A, B) (*p <* 0.05; d = 5.9 versus control). On the contrary, cells treated with AS2034178 demonstrated significant wider wounds than control cells after 24 h (*p <* 0.01; d = 5.9 versus control) (Fig. 3A, B), suggesting that FFA1 agonism inhibits cell migration. To confirm this, the effect of AS2034178 was examined in the presence of GW1100 and indeed, the antagonist completely blocked the negative effects of the agonist on migration and facilitated wound healing that approached the effects of PMA after 24 h (*p <* 0.01; d = 4.7 versus AS2034178 alone) (Fig. 3A, B). Interestingly, GW1100 alone caused nearly full closure of the wound after 24 h, and together, these data suggest that FFA1 agonism negatively regulates cell migration.

To confirm this hypothesis, we performed in vitro transwell cell migration assays and our results show that cells treated for 16 h with AS2034178 exhibited significantly lower migration towards the serum chemoattractant in the lower chamber compared to vehicle-treated controls (*p <* 0.01; d = 11.1 versus control) (Fig. 3C, D). On the contrary, cells treated with the FFA1 antagonist GW1100 alone demonstrated significantly higher migration compared to control (*p <* 0.01; d = 2.7 versus control), and importantly, GW1100 inhibited the negative regulation of migration induced by AS2034178 (*p <* 0.01; d = 6.7 versus AS2034178) (Fig. 3C, D). Collectively, the results of our wound healing and transwell migration assays demonstrate that FFA1 agonism can negatively modulate the migratory capacity of ACHN RCC cells.

### FFA1 regulates invasion through the basement membrane

Given our results showing that FFA1 regulates pRCC cell migration, we assessed the role of the receptor in modulating the invasion of pRCC cells into and through the basement membrane matrix as a measure of metastasis. Here, we utilized Matrigel transwell assays and our results demonstrate that cells treated for 24 h with AS2034178 exhibited significantly lower invasion through the extracellular Matrigel matrix, compared to vehicle-treated controls (*p <* 0.01; d = 17.3 versus control) (Fig. 3E, F), suggesting that similar to migration, FFA1 agonism negatively regulates invasive properties of pRCC cells. Meanwhile, cells treated with GW1100 alone demonstrated significantly higher invasion compared to control cells (*p <* 0.01; d = 8.0 versus control), and again, GW1100 inhibited the negative regulation of invasion induced by AS2034178 to increase invasion (*p <* 0.01; d = 15.6 versus AS2034178) (Fig. 3E, F). These results validate that FFA1 agonism negatively modulates migratory and invasive properties of pRCC cells.

### FFA1-mediated pRCC cell proliferation is regulated by PI3K/AKT, NF-κB, and COX-2

**Fig. 4:**
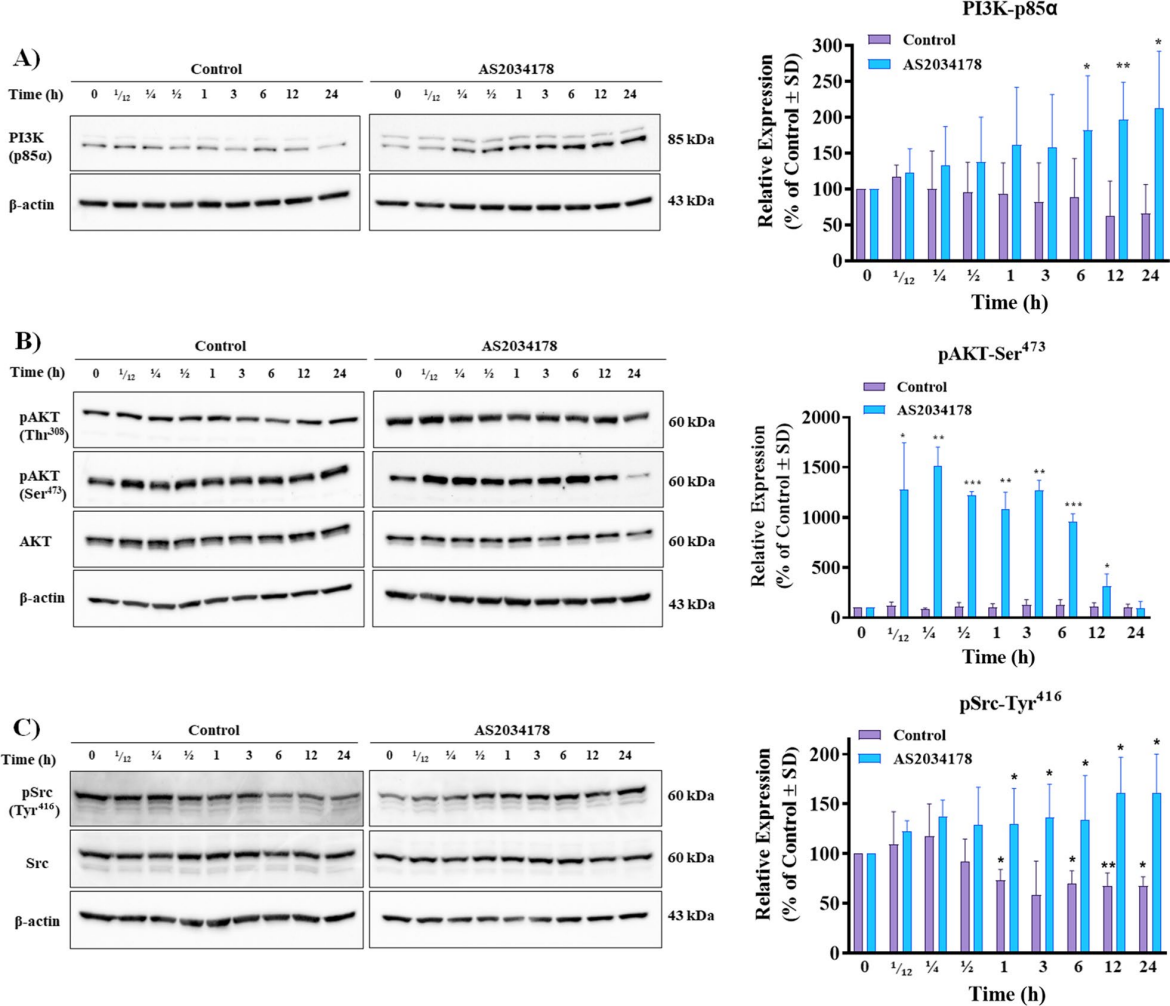
FFA1-mediated pRCC cell proliferation is regulated by PI3K/AKT and c-Src. **A** FFA1 agonism increased the expression of PI3K compared to the vehicle-treated control. The graphical results shown are representative of 5 independent experiments quantifying the p85α-PI3K subunit. **B** FFA1 agonism increased the phosphorylation of AKT at Ser^473^ compared to vehicle-treated control. The graphical results shown are representative of 5 independent experiments quantifying the phosphorylation of AKT at Ser^473^. **C** FFA1 agonism increased the expression of phosphorylated Src (Tyr^416^) compared to vehicle-treated control. The graphical results shown are representative of three independent experiments quantifying the phosphorylation of Src at Tyr^416^

GPCRs are significant upstream regulators of PI3K/AKT signaling cascades that are known to dictate crucial RCC processes including cell proliferation, migration, and survival [23]. Therefore, we probed the role of FFA1 PI3K/AKT signaling in pRCC cells and our results show that agonism of FFA1 with AS2034178 induced a time-dependent increase in expression of the p85α subunit of PI3K, compared to vehicle-treated control, with statistically significant increases across all *n* performed observed at 6, 12, and 24 h (Fig. 4A) (*p <* 0.05; d = 1.7, *p <* 0.01; d = 3.5 and *p <* 0.05; d = 2.9 versus vehicle-treated control, respectively). AKT is a downstream effector of PI3K and upon its phosphorylation, AKT can regulate an array of oncogenic processes through the activation of various substrates [23, 24]. Agonism of FFA1 did not affect phosphorylation of AKT at Thr^308^ compared to vehicle-treated control (Fig. 4B), however, significant increases in phosphorylation of AKT at Ser^473^ were seen upon agonism of FFA1 from 5 min to 12 h following addition of AS2034178 (Fig. 4B).

Since engagement of the PI3K/AKT pathway by GPCRs can be mediated by the proto-oncogene *c-Src*, which exerts pleiotropic effects on cell proliferation and survival [25, 26], we also investigated the role of AS2034178 in *c-Src* activity. Consistent with its role, prolonged serum-starvation resulted in decreased phosphorylation of *c-Src* at Tyr^416^ at 1, 6, 12 and 24 h (*p <* 0.05; d = 3.5, *p <* 0.05; d = 3.3, *p <* 0.01; d = 55.4, and *p <* 0.05; d = 4.9 versus control, respectively) (Fig. 4C). Treatment with AS2034178 not only prevented this decrease, but significantly increased phosphorylation of *c-Src* at 1, 3, 6, 12, and 24 h (*p <* 0.05; d = 2.2, *p <* 0.05; d = 2.3, *p <* 0.05; d = 1.9, *p <* 0.05; d = 4.0, and *p <* 0.05; d = 3.3 versus control, respectively) (Fig. 4C). These data demonstrate that FFA1 agonism modulates *c-Src* signaling in ACHN pRCC cells, and suggest that *c-Src* may be the upstream mediator of PI3K/AKT activity, as described by others [26].

**Fig. 5:**
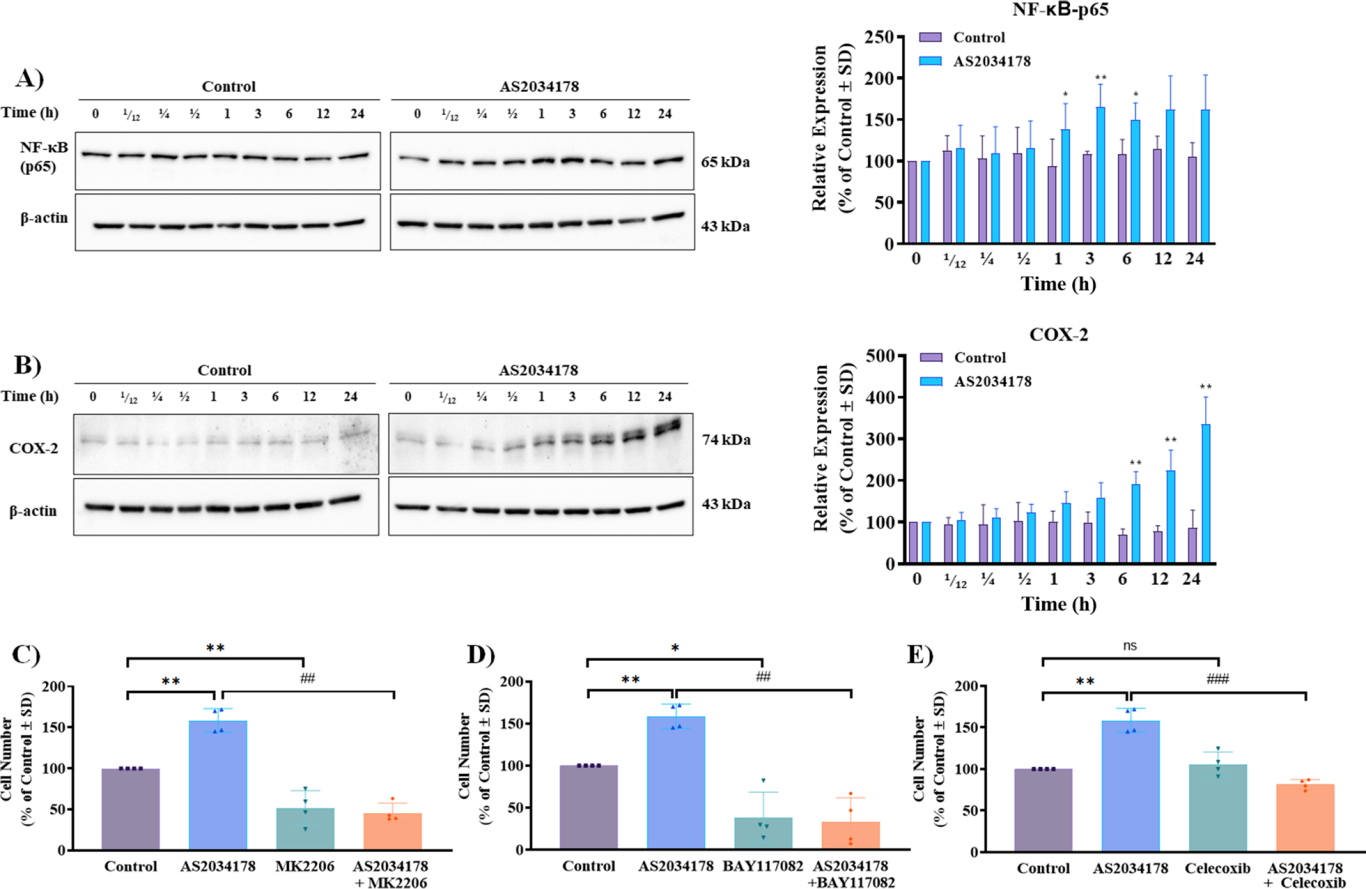
FFA1-mediated pRCC cell proliferation is regulated by AKT, NF-κB, and COX-2. **A** FFA1 agonism increased the expression of the p65 subunit of NF-κB compared to vehicle-treated control. The graphical results shown are representative of four independent experiments quantifying the p65 subunit. **B** FFA1 agonism increased the expression of COX-2 compared to vehicle-treated control. The graphical results shown are representative of four independent experiments quantifying COX-2. Statistical significance was determined by paired t-test. * denotes *p *< 0.05, **denotes *p* < 0.01, and *** denotes *p* < 0.001 versus the control condition. **C** MK2206 (1 µM) significantly decreases FFA1-mediated cell proliferation (*p* < 0.01; d = 8.35, versus AS2034178). When used alone, MK2206 inhibited serum-induced cell proliferation compared to control condition (*p* < 0.01; d = 3.25, versus control). **D** BAY 11-7082 (1 µM) significantly inhibited cell proliferation in the presence of AS2034178 (*p* < 0.01; d = 5.56, versus AS2034178). Compared to the vehicle-treated control condition, BAY 11-7082 suppressed serum-induced pRCC cell proliferation (*p* < 0.05; d = 2.90, versus control). **E** Celecoxib decreases FFA1-mediated cell proliferation (*p* < 0.001; d = 6.70, versus AS2034178). Statistical significance was determined by paired *t*-test. Graphs depict combined replicate data from four independent experiments each performed in triplicate. *denotes *p* < 0.05, and ** denotes *p* < 0.01 versus the control condition, while ## denotes *p* < 0.01, and ### denotes *p*< 0.001 versus the AS2034178-treated condition

Since PI3K/AKT signaling causes nuclear translocation and transcriptional activity of NF-κB through downstream activation of the inhibitor of nuclear factor-κB (IκB) kinase (IKK) complex, resulting in the inhibition of apoptosis and promotion of tumor growth in cancer models [26–28], we next assessed the effects of FFA1 agonism on activation of the p65 subunit of NF-κB as a measure of its activity. Agonism with AS2034178 led to a statistically significant increase in the expression of NF-κB p65 at 1, 3, and 6 h after treatment (*p <* 0.05; d = 1.4, *p <* 0.01; d = 2.1, and *p <* 0.05, d = 2.2 versus vehicle-treated control, respectively) (Fig. 5A), demonstrating that FFA1 regulates NF-κB in pRCC cells. Transcriptional activity of NF-κB directly upregulates expression of cyclooxygenase-2 (COX-2), amongst other mediators, which can induce cell proliferation [29] and accordingly, our data demonstrate that FFA1 agonism drastically induces COX-2 expression at 6, 12, and 24 h (*p <* 0.01; d = 5.2, *p <* 0.01; d = 4.2, and *p <* 0.01; d = 4.6 versus vehicle-treated control, respectively) compared to vehicle-treated controls (Fig. 5B). Together, these data suggest that FFA agonism activates signaling though *c-Src*/PI3K/AKT/NF-κB/COX-2 in ACHN pRCC cells.

Since our results show that FFA1 agonism increases pRCC cell proliferation and leads to the activation of PI3K, AKT, NF-κB, and COX-2 expression, we next sought to investigate the role of the AKT/NF-κB/COX-2 pathway on FFA1-induced cell proliferation. Here, we utilized the AKT inhibitor MK2206 [30], the IκB-α/IKK inhibitor BAY 11-7802 [31], which prevents NF-κB activity, and the COX-2 selective inhibitor celecoxib [32], to assess their effects on AS2034178-induced cell proliferation. When used alone, MK2206 (1 µM) significantly suppressed cell proliferation compared to vehicle-treated control (*p <* 0.01; d = 3.3 versus control), consistent with its inhibition of AKT (Fig. 5C). In the presence of AS2034178, MK2206 fully inhibited the AS2034178-mediated serum-induced cell proliferation effect, reducing it by approximately 70% (*p <* 0.01; d = 8.5 versus AS2034178) (Fig. 5C). Similarly, cells treated with BAY 11-7802 (1 µM) alone demonstrated significantly reduced cell proliferation compared to control cells (*p <* 0.05; d = 2.9 versus control), while BAY 11-7802 fully inhibited AS2034178-induced cell proliferation, reducing it by nearly 80% (p < 0.01; d = 5.6 versus AS2034178) (Fig. 5D). Since COX-2 expression is induced by NF-κB, we hypothesized that COX-2 inhibition would also significantly alter FFA1-induced proliferation, and indeed, celecoxib (0.5 µM) also significantly decreased AS2034178-induced cell proliferation (*p <* 0.001; d = 6.7 versus AS2034178), yet, had no effect alone (Fig. 5E). Together, our results demonstrate that FFA1-mediated serum-induced cell proliferation in pRCC cells is regulated by AKT, NF-κB, and COX-2 signaling.

### FFA1-mediated pRCC cell invasion is regulated by EGFR and ERK

**Fig. 6:**
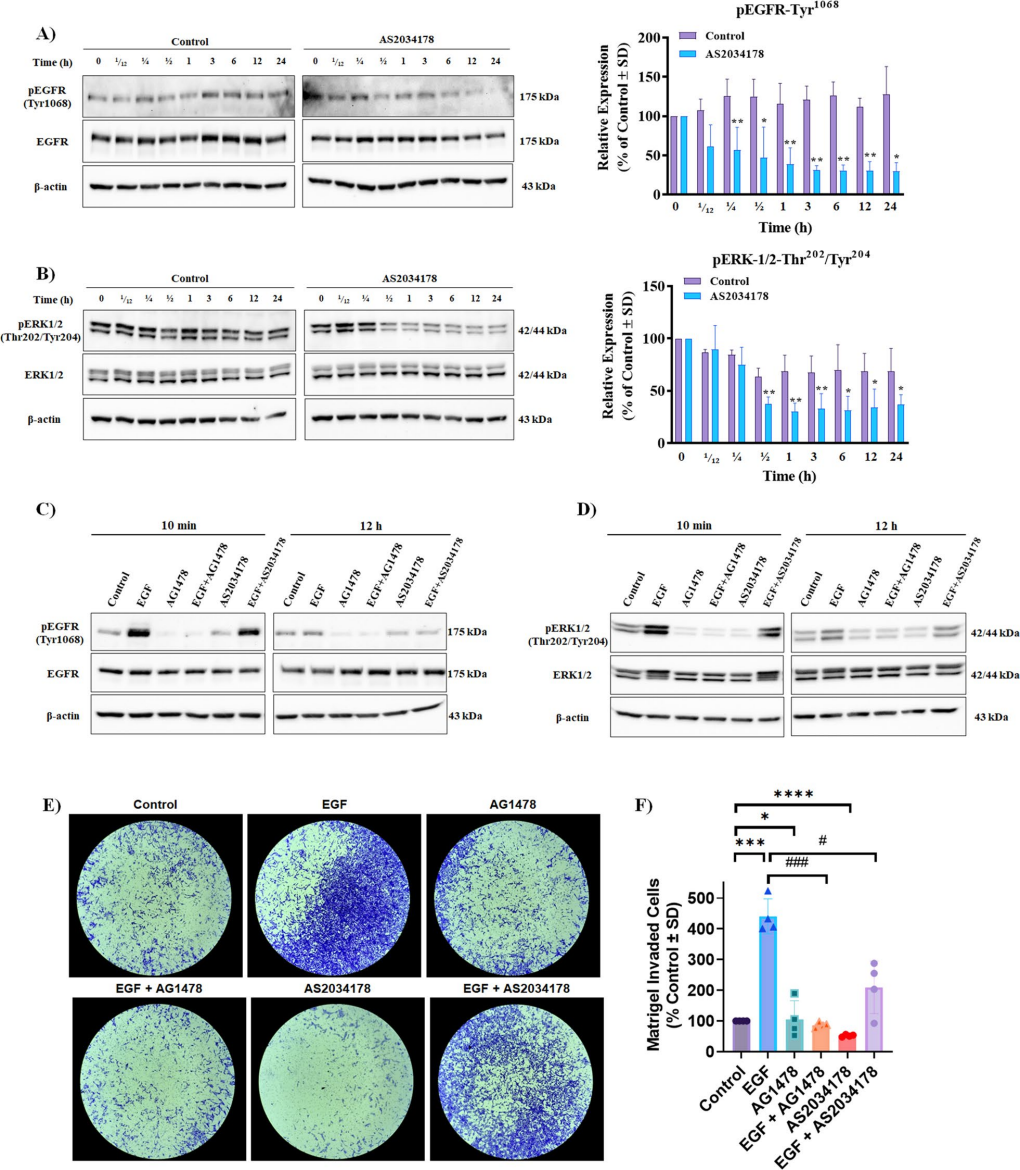
FFA1 regulates pRCC cell invasion through inhibition of EGFR and ERK. **A** FFA1 agonism inhibits phosphorylation of EGFR at Tyr^1068^ compared to vehicle-treated control. The graphical results shown are representative of three independent experiments quantifying phosphorylation of EGFR at Tyr^1068^. **B** FFA1 agonism inhibits phosphorylation of ERK1/2, respectively, compared to vehicle-treated control. The graphical results shown are representative of three independent experiments quantifying phosphorylation of ERK1/2. **C**, **D** FFA1 agonism inhibits EGF-induced EGFR phosphorylation and ERK1/2 phosphorylation. The results shown are representative of two independent experiments. Statistical significance was determined by paired* t*-test. * denotes *p* < 0.05 and ** denotes *p* < 0.01 versus the control condition. **E**, **F** EGF (10 ng/mL) stimulated significantly higher invasion through the extracellular Matrigel matrix compared to vehicle-treated controls (*p* < 0.001; d = 8.5, versus control). Meanwhile, cells treated with AG1478 (500 nM) alone demonstrated significantly lower invasion compared to control cells (*p* < 0.05; d = 1.5, versus control), and again, AG1478 inhibited the positive regulation of invasion induced by EGF to decrease invasion (*p* < 0.001; d = 8.7, versus EGF). AS2034178 (10 μM) significantly reduced the invasion of pRCC cells through the matrix (*p *< 0.0001; d = 17.7, versus control) and AS2034178 also partially inhibited the positive regulation of EGF-induced invasion (*p* < 0.05; d = 3.2, versus EGF). Statistical significance was determined by paired *t*-test. Graphs depict combined replicate data from four independent experiments each performed in triplicate. * denotes *p* < 0.05, *** denotes *p* < 0.001, and **** denotes *p* < 0.0001 versus the control condition, while ### denotes *p* < 0.001 versus the EGF-treated condition and # denotes *p* < 0.05 versus the AS2034178-treated condition

EGFR, a member of the RTK superfamily, is frequently expressed in all subtypes of RCC, and its expression is heightened in up to 60% of RCC tissues [33]. Given the prevalent role of GPCRs in EGFR transactivation, we sought to investigate the effects of FFA1 agonism on the activation of EGFR. Agonism with AS2034178 significantly reduced phosphorylation of EGFR at Tyr^1068^ an effect that was seen in as fast as 5 min, and was statistically lower than control at every time point from 15 min to 24 h (15 min: *p <* 0.01; d = 2.7, 30 min: *p <* 0.05; d = 2.5, 1 h: *p <* 0.01; d = 3.3, 3 h: *p <* 0.01; d = 6.9, 6 h: *p <* 0.01; d = 7.1, 12 h: *p <* 0.01; d = 7.4, and 24 h: *p <* 0.05; d = 3.7, versus vehicle-treated control, respectively) (Fig. 6A), demonstrating that FFA1 agonism negatively regulates EGFR in pRCC cells.

Since activation of the ERK1/2 MAP kinases is a well-described downstream mediator of both GPCR and EGFR activity and is known to contribute significantly to human primary cancers and tumor-derived cell line carcinogenesis, we investigated the activity of ERK1/2, as a function of its phosphorylation, following agonism of FFA1 with AS2034178. Agonism of FFA1 significantly decreased phosphorylation of ERK1/2 at 0.5, 1, 3, 6, 12 and 24 h after treatment, relative to that of vehicle-treated control (*p <* 0.01; d = 37., *p <* 0.01; d = 3.1, *p <* 0.01; d = 2.3, *p <* 0.05; d = 2.0, *p <* 0.05; d = 2.0, and *p <* 0.05; d = 2.0 versus vehicle-treated control, respectively) (Fig. 6B), suggesting negative modulation of ERK1/2 by FFA1 in pRCC cells.

The transactivation of EGFR by GPCR agonism is well-described and known to cause downstream activation of MEK/ERK1/2 MAP kinase pathways, which in turn influence invasion and migration capacities of tumor cells [34–37]. Given our results, we hypothesized that FFA1 suppresses pRCC cell invasion via inhibition of EGFR-mediated ERK1/2 signaling in pRCC cells. As expected, EGF (10 ng/mL) readily facilitates phosphorylation of both EGFR and ERK1/2 in pRCC cells after both short (10 min) and long-term (12 h) exposure, with the shorter effect being more robust, indicative of transient activation of EGFR by its cognate mitogen over time (Fig. 6C). The selective EGFR inhibitor AG1478 (500 nM) [38] blocked the effects of EGF at both time points (Fig. 6C). As was shown in Fig. 6A, treatment with AS2034178 alone inhibited phosphorylation of EGFR compared to vehicle-control (Fig. 6C). In the presence of EGF, the FFA1 agonist significantly decreased, but did not fully block, the EGF-induced autophosphorylation of EGFR at both 10 min and 12 h, demonstrating that FFA1 negatively regulates EGFR in a ligand-independent manner (Fig. 6C). Moreover, EGF (10 ng/mL) robustly increased ERK1/2 phosphorylation after 10 min, and does so more moderately after 12 h in pRCC cells, and these effects were fully blocked by AG1478, confirming that EGF-mediated EGFR activation triggers downstream ERK1/2 signaling in pRCC cells (Fig. 6D). Importantly, treatment with AS2034178 again partially blocked the EGF-induced ERK1/2 phosphorylation, demonstrating a significant role for FFA1 in negative regulation of EGFR signaling to MAPK (Fig. 6D).

Next, we wished to determine if the FFA1-EGFR crosstalk modulates invasion of pRCC cells. Using our Matrigel invasion assay described above, our results unsurprisingly demonstrate that cells treated for 24 h with EGF (10 ng/mL) exhibited significantly higher invasion through the extracellular matrix compared to vehicle-treated controls (*p <* 0.001; d = 8.5 versus control) (Fig. 6E, F), confirming that EGFR is a crucial driver of pRCC cell invasion. Meanwhile, cells treated with AG1478 (500 nM) alone exhibited significantly lower invasion compared to control cells (*p <* 0.05; d = 1.5 versus control), and again, AG1478 inhibited the positive regulation of invasion induced by EGF to decrease invasion (*p <* 0.001; d = 8.7 versus EGF) (Fig. 6E, F). Agonism of FFA1 by AS2034178 significantly reduced the invasion of pRCC cells through the matrix (*p <* 0.0001; d = 17.7 versus control), which is consistent with our results obtained from Fig. 3A, B (Fig. 6E, F), however, AS2034178 also partially inhibited the positive regulation of EGF-induced invasion (*p <* 0.05; d = 3.2 versus EGF), demonstrating that at least in part, FFA1 negatively regulates pRCC cell invasion through inactivation of EGFR signaling (Fig. 6E, F).

### FFA1 inhibits STAT3 activity independent of EGFR and β-arrestin-2

**Fig. 7:**
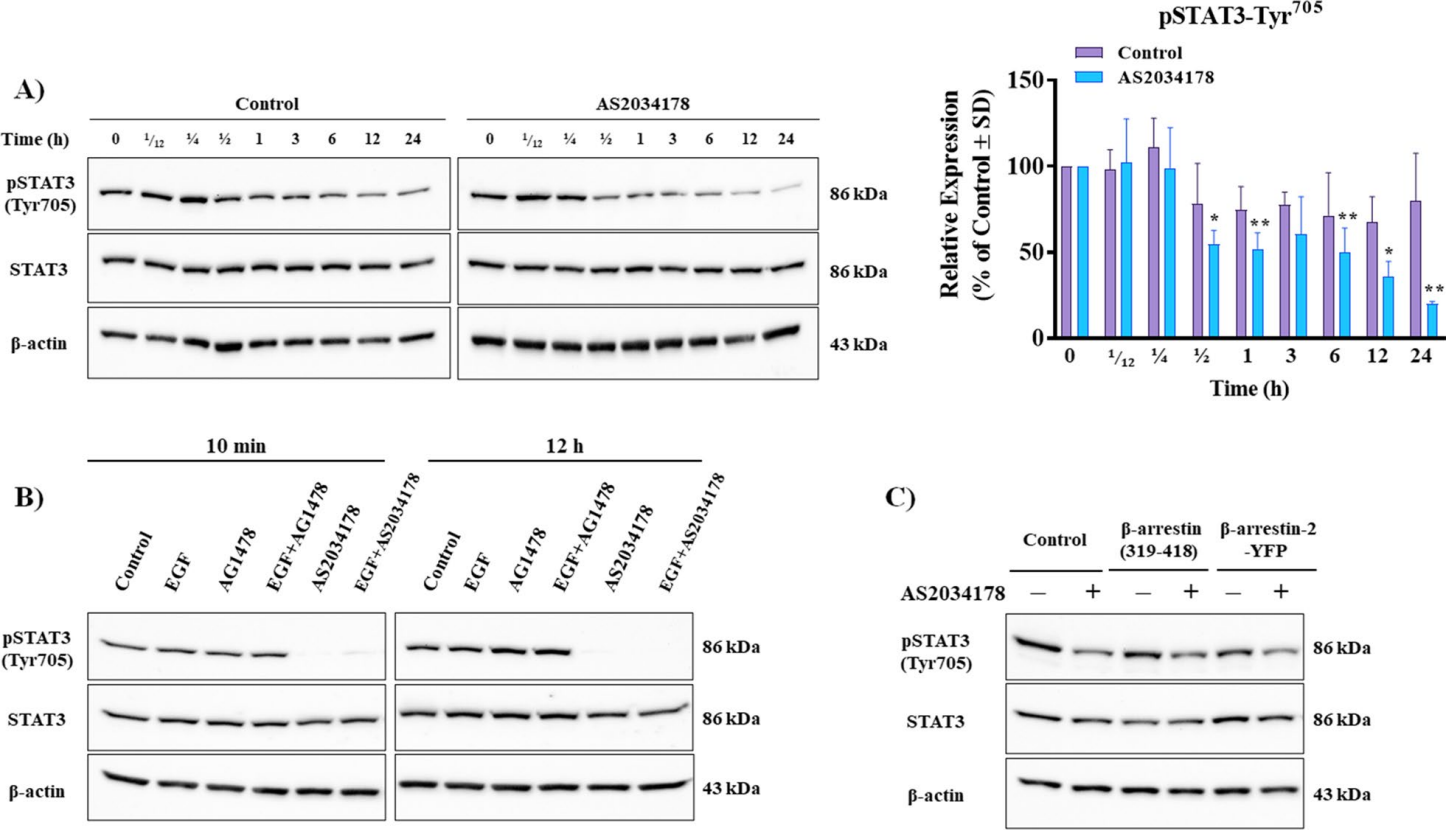
FFA1 inhibits phosphorylation of STAT3 independent of EGFR and β-arrestin 2. **A** FFA1 agonism inhibits phosphorylation of STAT3 at Tyr^705^ compared to vehicle-treated control. The graphical results shown are representative of three independent experiments quantifying phosphorylation of STAT3 at Tyr^705^. Statistical significance was determined by paired *t*-test. * denotes *p* < 0.05 and ** denotes *p* < 0.01 versus the control condition. **B** FFA1 agonism inhibits STAT3 phosphorylation independent of EGFR. **C** FFA1 agonism inhibits STAT3 phosphorylation independent of β-arrestin-2. Serum‐starved ACHN cells were seeded in 6-well plates and transfected with pcDNA3-β-arrestin (319-418) and pcDNA3-β-arrestin-2-YFP. After 24 h, cells were incubated with AS2034178 (10 μM) for another 24 h, and the lysate was collected. The results shown are representative of three independent experiments.

STAT3 is a cytoplasmic transcription factor responsible for transcription of a myriad of genes involved with the cell cycle, apoptosis, and migration and STAT3 activation in responses to cytokines and upstream influences like EGFR, plays important roles in genitourinary cancers, including RCC [39–44]. Agonism of FFA1 with AS2034178 significantly decreased phosphorylation of Tyr^705^ of STAT3 at 30 min, 1 h, 6 h, 12 and 24 h compared to vehicle-treated control, respectively (*p <* 0.05; d = 0.8; *p <* 0.01; d = 2.0, *p <* 0.01; d = 0.4, *p <* 0.05; d = 2.6, and *p <* 0.01; d = 3.0 versus vehicle-treated control, respectively) (Fig. 7A). Given that our results showing that FFA1 agonism inhibits phosphorylation of both EGFR and STAT3 in pRCC cells, we wanted to examine whether FFA1 prevents STAT3 phosphorylation via inhibition of EGFR. Our results reveal that EGF (10 ng/mL) did not induce STAT3 phosphorylation in pRCC cells compared to vehicle control at either 10 min or 12 h timepoints (Fig. 7B), and moreover, AG1478 (500 nM) did not affect STAT3 phosphorylation either alone or in the presence of EGF, suggesting that in ACHN pRCC cells, EGFR is not an upstream regulator of STAT3 (Fig. 7B). On the other hand, AS2034178, whether in the absence or presence of EGF, strongly inhibited phosphorylation of STAT3 at both 10 min and 12 h (Fig. 7B), indicating that FFA1 regulates STAT3 independent from EGFR in pRCC cells.

β-Arrestin is a GPCR partner protein whose functions as a GPCR signal scaffolding protein are well-described [45], and FFA1 is also known to signal through β-arrestins [46, 47]. Since β-arrestin seems to also play a role in scaffolding to STAT3 activation [48–50], we examined if the FFA1-induced inhibition of STAT3 involved β-arrestins. To do so, we overexpressed ACHN pRCC cells with either β-arrestin-2-YFP or β-arrestin-(319-418), which acts as a dominant-negative mediator of arrestin function [51]. As illustrated in Fig. 7C, there was no appreciable difference in the effects of FFA1 agonism on STAT3 phosphorylation upon overexpression of either β-arrestin-2 or β-arrestin-(319-418), suggesting that the effects of FFA1 on STAT3 are not dependent on β-arrestins, and are likely then mediated by G-protein signals. To ensure that this result was not simply due to lack of expression of the β-arrestin transfectants, we detected the expression of the YFP tag by immunoblot (data not shown).

### FFA1 agonism regulates EMT

**Fig. 8:**
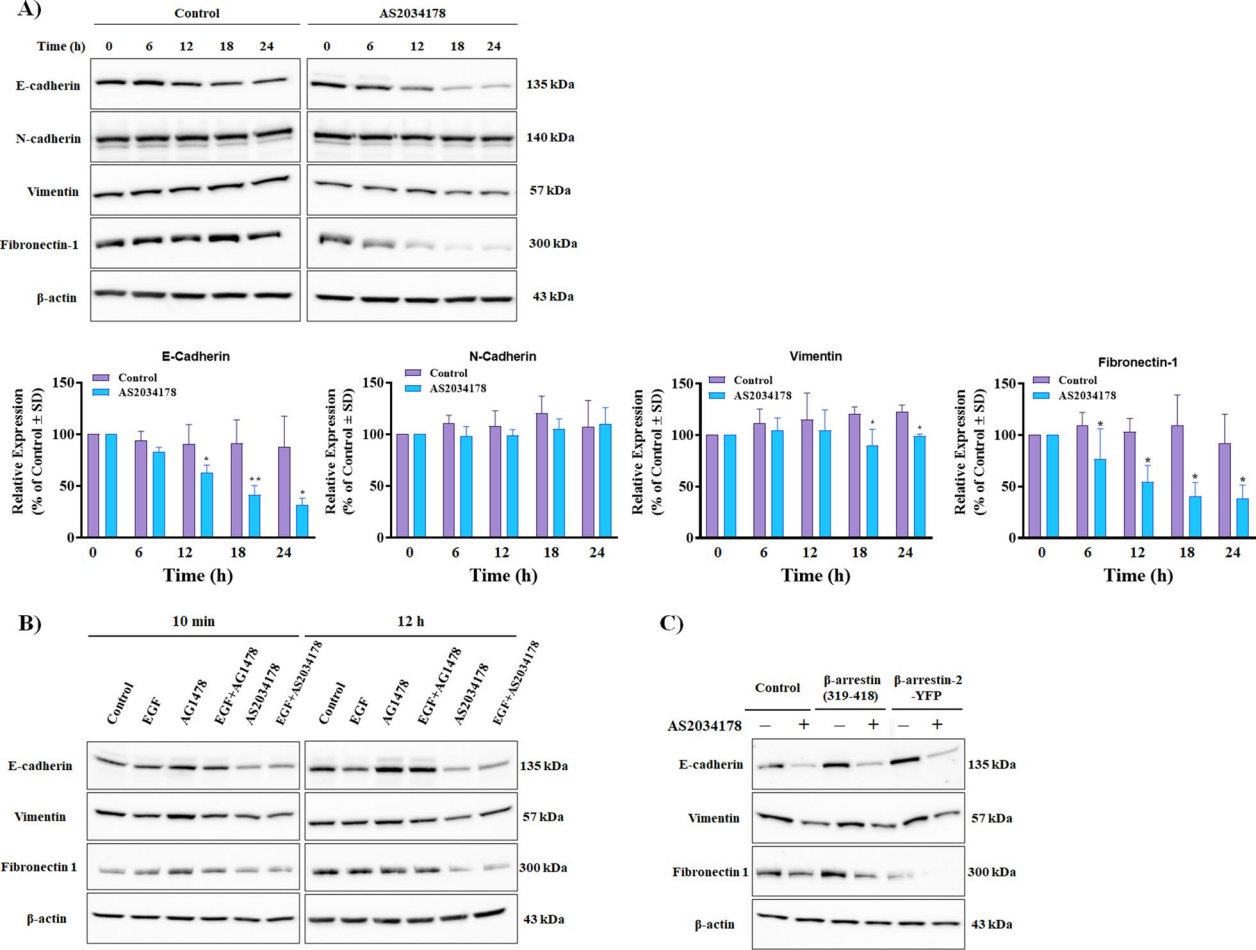
FFA1 agonism modulates regulators of EMT. **A** FFA1 agonism inhibits the expression of E-cadherin, vimentin, and FN1 compared to vehicle-treated control. The graphical results shown are representative of four independent experiments quantifying the respective protein. Statistical significance was determined by paired *t*-test. * denotes *p* < 0.05 and ** denotes *p* < 0.01 versus the control condition. **C** Downregulation of β-arrestin results in increased expression of FN1 whereas upregulation of β-arrestin-2 decreases FN1 expression. FFA1 agonism exhibited strong activation of FN1 when β-arrestin-2 was overexpressed and minimal activation when β-arrestin was downregulated. The results shown are representative of three independent experiments

Epithelial-mesenchymal transition (EMT) is a hallmark of migration that allows polarized epithelial cells to acquire a stem-cell like mesenchymal phenotype, which contributes to cancer metastasis [52–54]. EMT is commonly characterized as the loss of the epithelial marker E-cadherin and upregulation of the mesenchymal markers N-cadherin, vimentin, and fibronectin-1 (FN1) [55]. Thus, we investigated whether FFA1 is an upstream regulator of EMT in pRCC by gauging the effects of FFA1 agonism on E-cadherin, N-cadherin, vimentin, and FN1. Agonism of FFA1 with AS2034178 led to a significant decrease in the expression of E-cadherin at 12 h, 18 and 24 h compared to vehicle-treated control (*p <* 0.05; d = 1.9, *p <* 0.01; d = 2.9 and *p <* 0.01; d = 2.7 versus vehicle-treated control, respectively) (Fig. 8A). While FFA1 agonism did not affect the expression level of N-cadherin (Fig. 8A), a significant decrease in the expression of the mesenchymal marker vimentin was observed at 12 and 24 h after treatment (*p <* 0.05; d = 1.3, and *p <* 0.01; d = 0.5 versus vehicle-treated control, respectively) (Fig. 8A). Furthermore, FFA1 agonism strongly inhibited FN1 in pRCC cells, relative to that of vehicle-treated control at 6 h, 12 h, 18 h, and 24 h after treatment (*p <* 0.05; d = 1.5, *p <* 0.05; d = 2.0 and *p <* 0.05; d = 1.9, and *p <* 0.05; d = 1.3 versus vehicle-treated control, respectively).

Next, we investigated whether the FFA1-mediated effects on EMT markers involve EGFR. Compared to vehicle-treated controls, EGF nor AG4178, alone or in combination with EGF, did not significantly affect the expression of E-cadherin, vimentin, and FN1 in pRCC cells (Fig. 8B). On the other hand, AS2034178 significantly decreased the expression of E-cadherin, vimentin, and FN1, both in the absence as well as the presence of EGF (Fig. 8B), suggesting that FFA1 regulates EMT independent of EGFR in pRCC cells.

Finally, given the role of β-arrestins in migration and invasion of cancer cells [56–58] and their interaction of β-arrestin-2 with FFA1, we wished to examine the role of β-arrestin-2 in the regulation of EMT by FFA1 in pRCC cells. No discernable effects of FFA1 agonism on the expression of E-cadherin and vimentin were observed in cells overexpressing either β-arrestin-2-YFP or the β-arrestin dominant-negative mutant β-arrestin-(319-418), suggesting that β-arrestin is not involved in these processes (Fig. 8C). Interestingly, blockade of β-arrestin function in the presence of the dominant-negative increased the expression of FN1 in both untreated and AS2034178-treated cells, whereas overexpression of β-arrestin-2 significantly decreased the expression of FN1 in both cases, suggesting that β-arrestin signaling is required for the regulation of the EMT marker FN1 by FFA1 in pRCC cells (Fig. 8C).

### FFA1 agonism promotes pRCC in vivo tumor growth

**Fig. 9:**
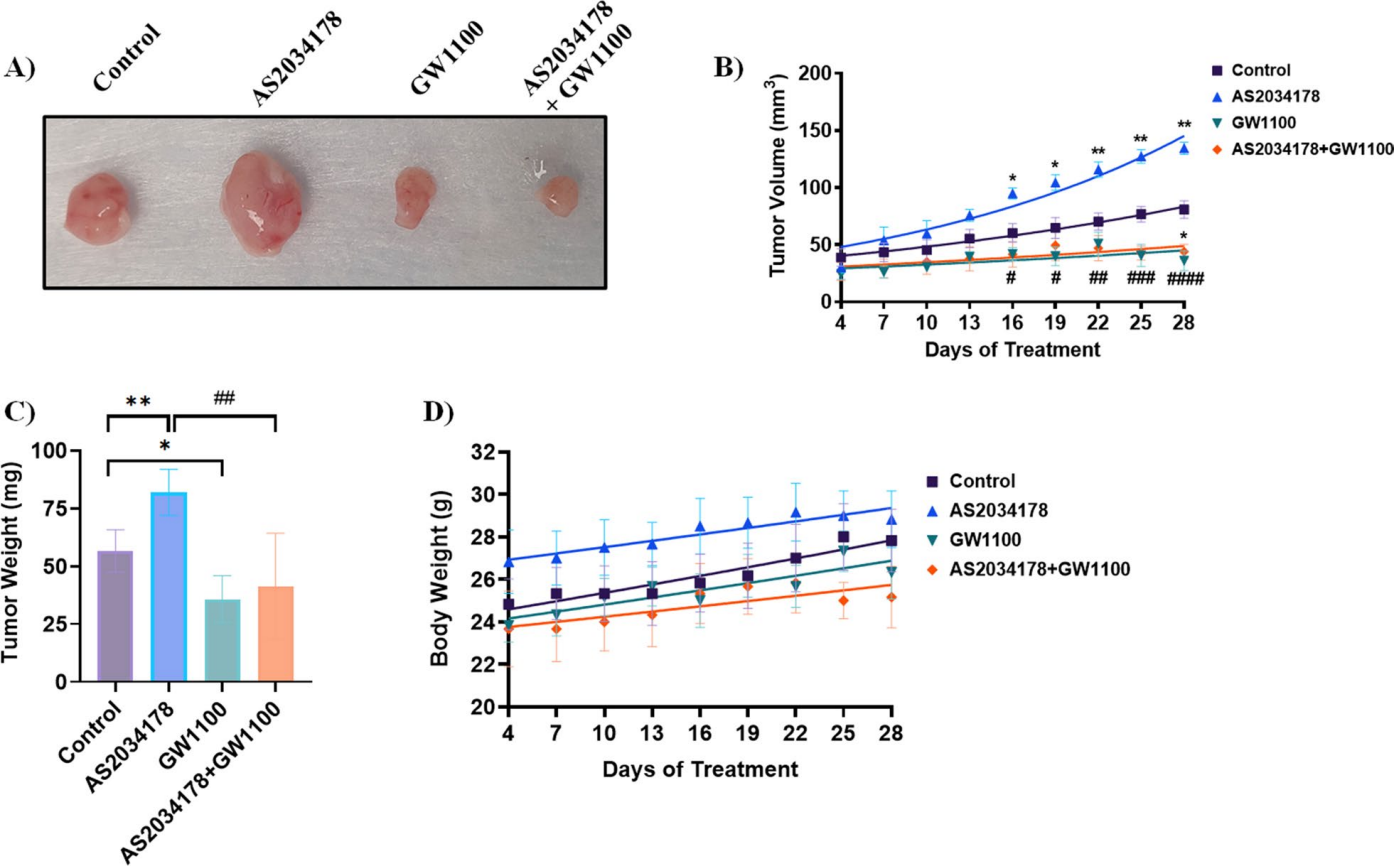
FFA1 agonism increased tumor growth in vivo. An ACHN cell-derived pRCC tumor xenograft model was established in athymic nude mice treated daily with intraperitoneal injections of vehicle, AS2034178 (10 mg/kg), GW1100, or a combination of AS2034178 + GW1100. Six male mice were used in each cohort and tumors were measured every 3 days for 28 days. Representative images of harvested tumors after 28 days of treatment are displayed (**A**). **A**, **B** Treatment with AS2034178 led to a significant increase in tumor size compared to the vehicle-control group. A statistically significant increase in tumor volume in AS2034178-treated mice was observed from day 16 onward. Mice treated with GW1100 alone showed a statistically significant reduction in tumor size versus vehicle-treated animals at day 16. The combination of AS2034178 and GW1100 caused a significant decrease in the AS2034178-induced tumor size, with statistical significance reached from day 16 onward. **C** After 28 days, tumors were excised and weighed, with AS2034178 treated animals demonstrating a significantly increased tumor mass versus vehicle-treated animals, while GW1100 treatment alone decreased tumor mass versus control. AS2034178-induced tumors exhibited significantly reduced tumor mass in the presence of GW1100. **D** To ensure that tumor reduction was not due to metabolic effects of treatment or tumor-induction, body weight was measured over the treatment period and showed no significant difference. Statistical significance was determined by two-way ANOVA. * denotes p < 0.05 and ** denotes p < 0.01 versus the vehicle-control condition, while # denotes p < 0.05, ## denotes p < 0.01, and #### denotes p < 0.0001 versus the AS2034178-treated condition

Due to the tumor-promoting effects of FFA1 signaling observed in vitro, we further investigated the effects of FFA1 signaling on pRCC tumor growth in vivo. Seven days following implantation of ACHN cell xenografts, mice were treated daily for 28 days with either vehicle, AS2034178 (10 mg/kg), GW1100 (10 mg/kg), or a combination of both AS2034178 and GW1100, and tumor volume was measured every three days. As shown in Fig. 9A, B, tumors derived from ACHN cells in mice treated with AS2034178 grew significantly larger than those from vehicle-control treated mice, beginning at day 16 of treatment, demonstrating that FFA1 agonism promotes tumor growth in the pRCC xenograft model. On the other hand, GW1100 treatment caused significant reductions in tumor growth in a time-dependent manner (Fig. 9A, B), with statistically significant reduction in tumor size compared to vehicle observed after 16 d (Fig. 9A, B). The growth promoting effects of AS2034178 were significantly inhibited by GW1100 beginning from day 16 on (Fig. 9A, B). Similarly, the average tumor weight was significantly higher in the AS2034178-treated group compared to the vehicle-treated control group (*p* < 0.01 vs. vehicle-control; d = 2.9), while on contrary, treatment with GW1100 alone or in the presence of AS2034178 led to significantly smaller tumor mass (*p* < 0.05 vs. vehicle-control, d = 2.2; *p* < 0.05 vs. AS2034178; d = 2.3, respectively) (Fig. 9C). To ensure that these reductions were not due to metabolic changes induced by treatment or tumor-induction, we assessed the body weight in each group during the treatment period and did not find a significant change between treatment groups, although the agonist group appeared to trend higher from the beginning (Fig. 9D). To account for differences in initial body weights that may have contributed to this, we assessed net percent change in maximal body weight over the study period and noted no discernible differences between groups (Additional file 1: Fig. S2) These observations are in agreement with our in vitro proliferation results indicating that FFA1 signaling accelerates pRCC tumor growth.

## Discussion

RCC comprises approximately 3% of all adult malignancies, with pRCC representing the most frequent non-clear cell subtype, accounting for 15–25% of all cases [3]. pRCC itself is highly heterogenous with distinct pathological classification and variable clinical prognosis, with metastatic pRCC exhibiting clinically worse outcomes than ccRCC [59]. Our results show that the free-fatty acid receptor FFA1, which is activated by a variety of medium-to-long chained FFA plays important roles in modulating pRCC cell behaviors including proliferation and migration/invasion. Our qualitative observations with clinical tissues suggest that FFA1 expression is markedly upregulated in both T1a and T3a pathological grades whereas patient-matched normal adjacent tissue show little expression of FFA1. While the lower stage represents a localized tumor of less than 7 cm in size, the higher stage is indicative of a more progressive disease with invasion through the renal vasculature or fat deposits. Interestingly, FFA1 expression was comparatively lower in human ccRCC tissue samples relative to that of adjacent normal tissue samples, and seemed to be lost as ccRCC progressed from T1 to T4. Since our results demonstrate up-regulation of FFA1 in advanced tumors and given that FFA1 agonism strongly inhibits migration and invasion, it is tempting to speculate that aberrant increases in receptor expression are a compensatory consequence of pRCC tumors and may suggest that FFA1 expression could be a potential biomarker and unique target for the treatment of advanced pRCC. A significant challenge towards development of RCC therapeutics centers on the vast heterogeneity of these cancers, even within the individual subtypes. For instance, papillary RCC alone can be divided into type 1 or type 2 based on distinct morphological and histological characteristics and a more recently recognized reverse polarity pRCC subtype has also recently been proposed [59–61]. Along these lines, pRCC is driven by genetic heterogeneity, with nearly ubiquitous chromosome 7 and 17 gains in type 1 pRCC, while type 2 pRCC commonly reflects gains in chromosomes 12, 16, and 20, and gains to chromosomes 2 and 3 have also been described [62, 63]. In addition to chromosomal gain, loss of chromosome 9p and intra-chromosomal rearrangement of chromosomes 1–3 have also been identified in pRCC [62, 64, 65], and together, these alterations reflect a broad expanse towards pathological variability. In addition to genetic heterogeneity, DNA methylation and epigenetic modifications also contribute to RCC variability [66]. Chromosomal alterations have been shown to directly impact many genes in pRCC including the protooncogene MET, which is common to type 1 pRCC, as well as cyclin-dependent kinase inhibitor 2A (CDKN2A) and downstream cell-cycle related proteins, including Hippo/YAP1, p53, and mTOR. Moreover, genes involved in chromatic and telomere structure and metabolism are also commonly mutated in pRCC, resulting in alterations of their downstream signal effectors [67]. On the contrary, ccRCC tends to be represented by characteristic VHL gene mutations (Chromosome 3) with significant increases in metastasis upon gains to chromosomes 1q, 7, 8, and 20, or losses to chromosomes 1p, 9, and 18. Interestingly, the FFA1 gene is localized to chromosome 19q13, which, to our knowledge is not represented as a major site of genetic alteration in either pRCC or ccRCC. However, the 5′-flank of the FFA1 gene contains a variety of conserved transcription factor binding sequences that readily regulate FFA1 expression, including AP2, STAT, IK2, PEA3, PBX, Bel1 and MEIS1, as well as the common transcriptional repressors AP4, ETS, RFX, EV1, ELK1, GATA [68], which we hypothesize are responsible for alteration of FFA1 expression in pRCC and ccRCC described here. Further experiments are underway in our laboratory to examine the precise mechanisms involved in the observed expression alterations.

Similar to that seen in tissues, our data demonstrate that FFA1 transcript is expressed in the human metastatic tumor-derived pRCC cell line ACHN but is lacking from the primary and metastatic ccRCC cell lines 786-O and Caki-1, respectively. While a limitation of the current study is the reliance on the ACHN line as a model of pRCC, it is important to note that other putative pRCC cell lines remain uncharacterized and contain non-characteristic mutations or are devoid of the characteristic c-MET mutations, and are also derived from non-metastatic primary tumors that do not readily form tumors in xenograft models, limiting their pRCC-specific utility [61, 69, 70]. Meanwhile ACHN cells, which were derived from pleural metastasis of RCC are highly migratory, lack ccRCC markers but demonstrate the hallmark characteristics of the papillary subtype, including the distinguishing c-MET mutation, and represent the most highly cited pRCC and third most highly cited RCC cell line [60, 69, 71]. Nonetheless, these results represent the initial foray of investigation of FFA1 in RCC and further work will be underway in our laboratory to validate these results in other pRCC tissue.

For the first time, we reveal that FFA1 activity regulates serum-induced proliferation of pRCC cells through engagement of the PI3K/AKT/NF-κB signaling pathway. While NF-κB activity is a well-known regulator of downstream transcription of a variety of inflammatory mediators including cytokines, interleukins, and TNF-α, it is also the chief inducer of COX-2 expression, which is known to be a key driver of RCC progression [72–75], prompting our investigation into COX-2 here. Consistent with this, our data demonstrate that FFA1 agonism facilitates robust COX-2 induction at 6–24 h following agonism, in accordance with the timetable of upstream activation of NF-κB, and the clinically available COX-2 inhibitor celecoxib fully inhibited the FFA1-mediated proliferative effects, suggesting that modulation of COX-2 may be of benefit towards pRCC proliferation. Interestingly, activation of the pro-oncogenic non-RTK tyrosine protein kinase c-Src is also robustly increased upon FFA1 agonism and notably, c-Src activity has also been shown to modulate FFA1 signals to proliferation of breast cancer cells [76], suggesting that c-Src may serve as a signaling intermediate between the receptor and PI3K/AKT. Further experiments are required to understand whether FFA1 interacts with c-Src directly or via other intermediaries to activate PI3K/AKT signaling in pRCC cells. Nonetheless, consistent with these molecular results, our in vivo data demonstrate significant growth and mass of pRCC xenografted tumors upon treatment with AS2034178 and moreover, our cell-based studies also translated to xenograft models that show that not only did the FFA1 inverse agonist block tumor growth induced by AS2034178, it also robustly decreased tumor growth on its own, confirming that FFA1 activity regulates proliferation and tumor size. Taken together, our results demonstrate that agonist-activated FFA1 increases activation of c-Src, that in turn activates downstream PI3K/AKT/NF-κB signaling and downstream COX-2 that increase cell proliferation.

Although FFA1 promotes pRCC cell proliferation and tumor growth, it negatively regulates cell motile activities including wound healing, migration, and invasion through extracellular matrix. Our data provide the first evidence of significant negative crosstalk between FFA1 and EGFR in pRCC cells, whereby FFA1 agonism robustly decreases EGFR phosphorylation and partially but significantly reduces EGF-mediated invasion, suggesting that FFA1 mediated reductions in invasibility are at least in part modulated by the receptors inhibitory influence on EGFR. While agonism of GPCRs, including FFA1, are well-known to increase phosphorylation of ERK1/2 via either G-protein or β-arrestin signaling, our results demonstrate a strong inhibitory effect of FFA1 agonism on ERK1/2 phosphorylation. Taken together with results showing inhibition of EFGR by FFA1 agonism, it is likely that the ERK1/2 inhibition we see here is a consequence of EGFR-mediated ERK1/2 activity, and our results with AS2034178 in the presence of EGF in these experiments (Fig. 6) support this conclusion. The manner by which FFA1 agonism inhibits EGFR activity in pRCC cells remain unclear and while it is possible that FFA1 agonism desensitizes signals utilized by EGFR, this would likely not directly decrease EGFR autophosphorylation but would rather likely be downstream of EGFR. FFA1-EGFR heteromerization or suppression of positive-crosstalk between other proteins and EGFR, as shown in prostate cancer cells for the related FFAR FFA4 [77], are also putative mechanisms. Similarly, the reasons for the partial blockade of EGF-mediated invasion by FFA1 agonism in pRCC cells remain unclear, although the relative expression of FFA1 compared to EGFR may be involved, as may the concentration of EGF used here (10ng/mL), which may be insurmountable by the relative degree of FFA1 agonism.

Interestingly, EGFR activation is a known regulator of the transcription factor STAT3, which itself is a key player in a myriad of cancers including RCCs [39–44]. While our results show significant suppression of STAT3 activity upon FFA1 agonism, this effect did not involve either EGFR nor β-arrestin-2, which itself is a negative regulator of STAT3 [78]. Together, these results make it likely that FFA1 signals to STAT3 independent of EGFR and via G proteins in these cells. Moreover, STAT3 activity has been suggested to induce MMP-9 expression, which can regulate RCC invasion [79], however, in our hands, FFA1 agonism had no effect on MMP-9 expression (data not shown). Taken together, our results postulate that FFA1 inhibits pRCC cell migration and invasion via inhibition of EGFR and STAT3, independent of each other.

Although there are a variety of reports, including our own in pRCC [15], on the involvement of the related FFAR FFA4 on EMT in cancers, to our knowledge the current report is the first to demonstrate the role of FFA1 in this process. Consistent with our findings that FFA1 agonism inhibits cell migration and invasion, FFA1 agonism also decreased expression of the EMT markers vimentin and FN-1. To our surprise, FFA1 agonism also robustly inhibited expression of E-cadherin in a time-dependent manner, a finding that is not in line with the traditional accepted view that loss of E-cadherin promotes mesenchymal phenotypes that promote cell migration. However, recent studies have challenged this dogma and shown that loss of E-cadherin in ovarian cancer cell lines decreases migration [80], while metastasized breast cancers exhibit greater expression of E-cadherin, or begin to re-express it during a reverting transition compared to primary cancer cells [81, 82]. Since FFA1 curiously decreases E-cadherin expression while at the same time inhibiting migration, and invasion, we hypothesize that ACHN cells continue to re-express E-cadherin for cell survival and to maintain EMT or partial EMT, while FFA1 opposes this process.

## Conclusions

In conclusion, this study is the first to delineate a significant role for FFA1 in papillary RCC cells and show upregulation of the receptor in pRCC tissue. Our results reveal significant, yet oppositional roles of FFA1 in these cells, whereby FFA1 agonism drives significant increases in cell proliferation and tumor growth, but strongly inhibits cell migration and invasion, which are indicative of metastasis. Given the importance of this receptor related to its activation by dietary fats and the known role of these fats in cancers including RCCs, further work is required to substantiate the role of FFA1 as a potential target for treatment of RCCs.

## Supplementary information

**Additional file 1: Figure S1.** FFA1 transcript and protein are expressed in ACHN pRCC cells. Expression of FFA1 mRNA transcript in ACHN, 786-O, and Caki-1 RCC cells by RT-PCR analysis. (−) represents the negative control condition with water in place of cDNA template, while (+) represents amplification of a template containing pcDNA3-FFA1-encoding plasmid, used as a positive control. The cell line indicated lane contains template cDNA derived from the respective cell line RNA followed by reverse transcription, while the -RT lane contains template cDNA derived from RNA lacking RT, to ensure that the resulting band was not a result of contaminating genomic DNA. GAPDH was used as the PCR-positive control. Expression of FFA1 protein in whole cell lysates of ACHN pRCC cells as detected by immunoblotting. Whole cell lysate was collected from serum-starved ACHN cells, HEK-293 cells, which lack FFA1 expression and serve as the negative control, and MCF-7 cells, which have previously been shown to express FFA1 and serve as a positive control. Representative data from both panels are shown from three independent experiments. **Figure S2. **Net percentage maximal body weight change. In order to account for differences in initial body weights, the maximal percent change in body weight was assessed and showed no significant difference between groups.

## Data Availability

All data generated or analyzed during this study are included in this published article or its associated supplementary information.

## Abbreviations

AKT: Protein kinase B
ccRCC: Clear cell renal cell carcinoma
COX-2: Cyclooxygenase-2
EGFR: Epidermal growth factor receptor
EMT: Epithelial–mesenchymal transition
ERK1/2: Extracellular signal regulated kinases-1/2
FN1: Fibronectin-1
FFA: Free-fatty acid
FFA1: Free-fatty acid receptor-1
FFA4: Free-fatty acid receptor-4
GPCR: G protein-coupled receptor
MAPK: Mitogen-activated protein kinase
MMC: Mitomycin C
PI3K: Phosphatidylinositol-3 kinase
PMA: Phorbol 12-myristate 13-acetate
pRCC: Papillary renal cell carcinoma
RCC: Renal cell carcinoma
RTK: Receptor tyrosine kinase
