# Supplementary material for: Free-fatty acid receptor-1 (FFA1/GPR40) promotes papillary RCC proliferation and tumor growth via Src/PI3K/AKT/NF-κB but suppresses migration by inhibition of EGFR, ERK1/2, STAT3 and EMT
Source: Cancer Cell Int. 2023 Jun 24;23:126. doi: 10.1186/s12935-023-02967-x (PMC10290327; doi:10.1186/s12935-023-02967-x)
Supplement: Supplementary file 1 — Additional file 1: Figure S1. FFA1 transcript and protein are expressed in ACHN pRCC cells. Expression of FFA1 mRNA transcript in ACHN, 786-O, and Caki-1 RCC cells by RT-PCR analysis. (−) represents the negative control condition with water in place of cDNA template, while (+) represents amplification of a template containing pcDNA3-FFA1-encoding plasmid, used as a positive control. The cell line indicated lane contains template cDNA derived from the respective cell line RNA followed by reverse transcription, while the -RT lane contains template cDNA derived from RNA lacking RT, to ensure that the resulting band was not a result of contaminating genomic DNA. GAPDH was used as the PCR-positive control. Expression of FFA1 protein in whole cell lysates of ACHN pRCC cells as detected by immunoblotting. Whole cell lysate was collected from serum-starved ACHN cells, HEK-293 cells, which lack FFA1 expression and serve as the negative control, and MCF-7 cells, which have previously been shown to express FFA1 and serve as a positive control. Representative data from both panels are shown from three independent experiments. Figure S2. Net percentage maximal body weight change. In order to account for differences in initial body weights, the maximal percent change in body weight was assessed and showed no significant difference between groups. [file 12935_2023_2967_MOESM1_ESM.docx]

**Additional File 1:**

**Karmokar and Moniri,** **Free-Fatty Acid Receptor-1 (FFA1/GPR40) promotes papillary RCC proliferation and tumor growth via Src/PI3K/AKT/NF-κB but suppresses migration by inhibition of EGFR, ERK1/2, STAT3 and EMT**


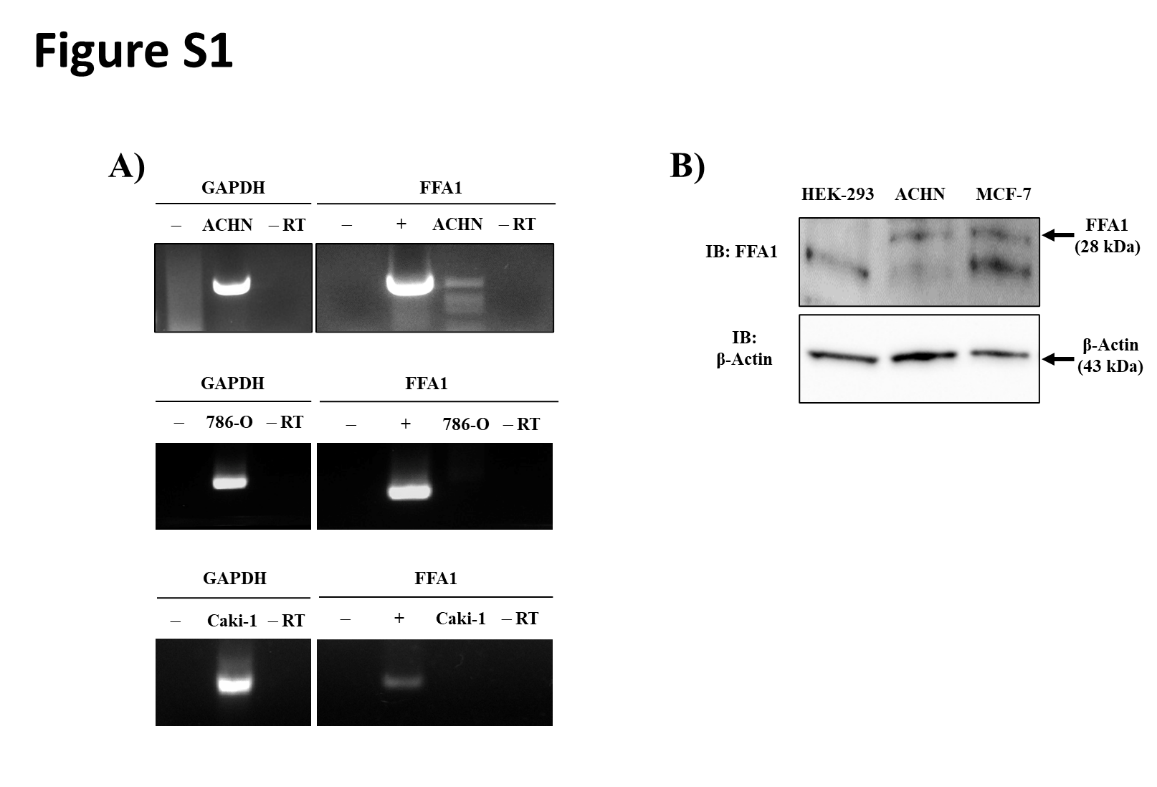


**Figure S1: FFA1 transcript and protein are expressed in ACHN pRCC cells.** (A) Expression of FFA1 mRNA transcript in ACHN, 786-O, and Caki-1 RCC cells by RT-PCR analysis. (−) represents the negative control condition with water in place of cDNA template, while (+) represents amplification of a template containing pcDNA3-FFA1-encoding plasmid, used as a positive control. The cell line indicated lane contains template cDNA derived from the respective cell line RNA followed by reverse transcription (RT), while the −RT lane contains template cDNA derived from RNA lacking RT, to ensure that the resulting band was not a result of contaminating genomic DNA. GAPDH was used as the PCR-positive control. (B) Expression of FFA1 protein in whole cell lysates of ACHN pRCC cells as detected by immunoblotting. Whole cell lysate was collected from serum-starved ACHN cells, HEK-293 cells, which lack FFA1 expression and serve as the negative control, and MCF-7 cells, which have previously been shown to express FFA1 and serve as a positive control. Representative data from both panels are shown from three independent experiments.

**
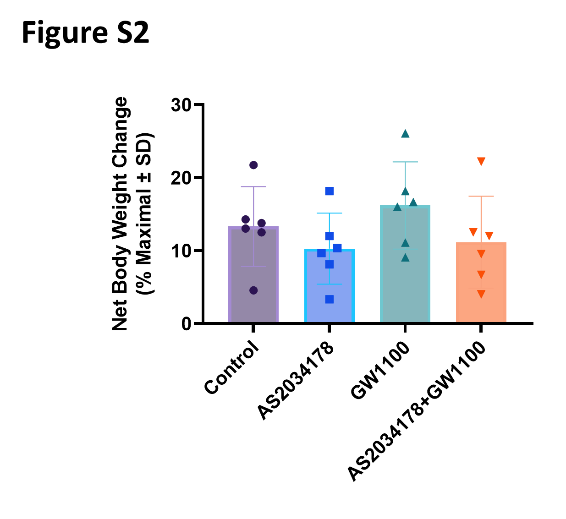
**

**Figure S2: Net percentage maximal body weight change.** In order to account for differences in initial body weights, the maximal percent change in body weight was assessed and showed no significant difference between groups.
